# Characterization of extracellular vesicles at parturition in dairy cows with late-gestation heat stress

**DOI:** 10.3168/jdsc.2025-0821

**Published:** 2025-10-30

**Authors:** L.T. Casarotto, H.N. Jones, L. Galio, C. Henry, P. Chavatte-Palmer, G.E. Dahl

**Affiliations:** 1 Department of Animal Sciences, University of Florida, Gainesville, FL 32611; 2 Department of Physiology and Aging, University of Florida College of Medicine, Gainesville, FL 32611; 3 Université Paris-Saclay, UVSQ, INRAE, BREED, Jouy-en-Josas, France, 78350; 4 Ecole Nationale Vétérinaire d'Alfort, BREED, Maisons-Alfort, France, 94700; 5 Université Paris-Saclay, INRAE, AgroParisTech, Micalis Institute, PAPPSO, Jouy-en-Josas, France, 78350

## Abstract

Extracellular vesicles (EV) are recognized as important mediators of cell-cell communication, capable of transferring various cargo between cells. Thus, EV are potential biomarkers of health status in heat-stressed cows. We hypothesized that heat stress during late gestation would alter the protein profile of EV at parturition in dairy cows. To test this, maternal plasma samples were collected within 2 h after parturition (n = 16–17/treatment) from cows exposed to late-gestation heat stress (HT) or an active cooling system (CL). The EV were isolated from plasma via single-step size exclusion chromatography. Preparations were checked for quality by transmission electronic microscopy and then quantified by nanoparticle tracking analysis before proteomic analysis. High-resolution MS was performed, and a two-sided, unpaired Welch's t-test was done on proteins showing at least 3 valid values in one group and at least 70% of valid values in the other group using log2 (label-free quantification intensity). The significance threshold was *P* ≤ 0.059, and proteins with a log2 fold change (CL/HT) above 1.2 or below 0.66 were considered significantly affected. Potential EV biomarkers from bovine plasma were detected, including several proteins significantly affected when comparing CL and HT. We noted more abundance in the CL group of laminin subunit gamma 1, α 2, and β 1; transferrin receptor protein 1; collagen IV; fibrinogen α chain, gamma-B chain, and β chain; von Willebrand factor; and SPN protein. Furthermore, in the CL group, the less abundant proteins included immunoglobulin domain lambda and heavy chains; HGF activator; protein HP-20 homolog; trafficking from endoplasmic reticulum to Golgi regulator (TFG) protein; amine oxidase 3; and collectin member 10. These findings suggest that these EV may play a role in modulating maternal metabolism and immune adaptations at the time of parturition within the maternal circulation. In addition, late-gestation heat stress alters the secretion of specific proteins that are involved in adaptations related to parturition. Further investigation is needed to better understand the functionality of EV during late gestation and the impact of heat stress on the molecules they carry.

## Highlights

• Heat stress during late gestation significantly alters EV content.
• Cooling increased extracellular matrix and coagulation proteins, perhaps for tissue repair and postpartum recovery.
• Cooling decreased immunoglobulin-related proteins and certain receptors in EV.
• Changes in EV proteins may reflect improved recovery and lower immune stress with cooling.

## Graphical Abstract

**Figure:**
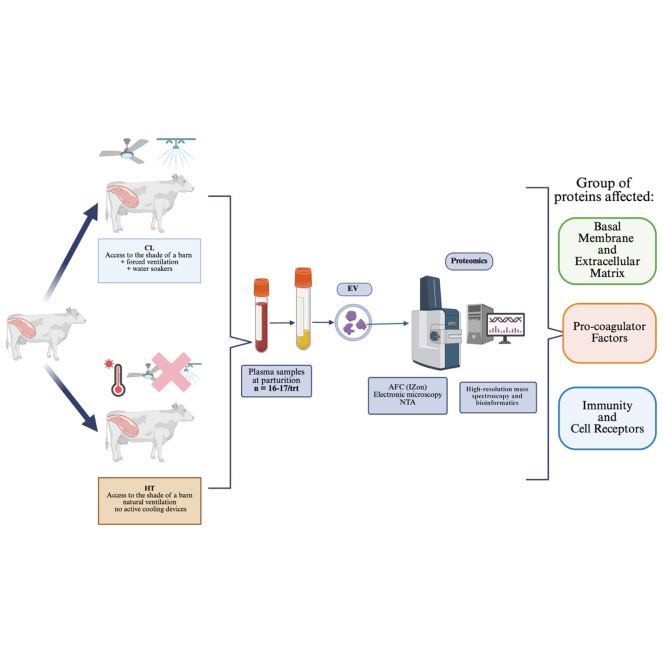
**Summary:** This study investigated how late-gestation heat stress (HT) affects extracellular vesicle (EV) protein profiles in dairy cows at parturition. Plasma EV were isolated from heat-stressed and cooled (CL) cows and analyzed by mass spectrometry. A total of 684 proteins were identified, of which 20 differed significantly between treatments. The EV from cooled cows were enriched in extracellular matrix and coagulation proteins, including laminins, collagen IV, fibrinogen, and von Willebrand factor. The presence of these proteins in CL cows suggests that cooling enhances molecular pathways involved in tissue repair and postpartum recovery. Conversely, immunoglobulin-related proteins and specific receptors were reduced in cooled cows, potentially reflecting lower immune stress. These findings indicate that HT during late gestation alters EV-mediated signaling related to metabolism, immune modulation, and tissue remodeling at parturition. The identified proteins may serve as potential biomarkers for assessing maternal adaptation and recovery, emphasizing the importance of environmental cooling during the dry period. NTA = nanoparticle tracking analysis; trt = treatment.

Environmental heat stress significantly affects dairy cows, leading to a range of negative outcomes. Specifically, it disrupts the cows' normal autonomic and behavioral thermoregulatory processes, which are vital for maintaining their body temperature in hot conditions (West, 2003). As a result, these animals often experience a decrease in DMI, which is crucial for their energy levels and milk production (Collier et al., 2017, 2019). Additionally, heat stress can lead to immunosuppression, making cows more vulnerable to infections and diseases, further compromising their overall health during the transition period (Molinari et al., 2022, 2023). Recently, several studies have documented these adverse effects, emphasizing that heat stress both affects the immediate well-being of lactating and dry dairy cows and has long-term implications for productivity and reproductive performance (Bernabucci et al., 2010; Tao and Dahl, 2013). Cows under heat stress may produce less milk (Tao et al., 2020), exhibit lower fertility rates (De Rensis and Scaramuzzi, 2003; Roth, 2020), and have longer recovery times from illness (Das et al., 2016; Soliman et al., 2025). Thus, addressing the challenges posed by environmental heat stress is critical to optimize animal health and performance.

Extracellular vesicles (**EV**) are diverse, nanosized membrane-bound structures produced by many cells throughout the body. These vesicles can be found in blood, urine, saliva, and milk (Koh et al., 2017). Extracellular vesicles are recognized for their capability to carry cargo that can alter cell phenotypes and facilitate long-distance communication between cells. This cargo includes mRNAs, microRNAs (**miRNAs**), lipids, proteins, and nucleic acids (Kalluri and LeBleu, 2020). Production of EV has been observed in numerous reproductive cells, such as follicular cells (da Silveira et al., 2012), oviductal cells (Al-Dossary et al., 2013; Almiñana et al., 2017), in vitro–produced embryos (Mellisho et al., 2017), endometrial cells (Ng et al., 2013; Burns et al., 2014), and the trophoblasts (Ortega et al., 2022; Tersigni et al., 2022). Extracellular vesicles contain miRNAs and proteins that regulate inflammatory responses and facilitate communication between cells within the placenta (Yang et al., 2019). Additionally, the placenta can interact with immune cells via EV, helping to balance immune activation and suppression throughout gestation. These studies emphasize the essential role of EV in various pathophysiological processes (Das and Kale, 2020; Nakahara et al., 2020) and highlight the growing focus on their potential to provide insights into maternal and fetal health throughout gestation and serve as indicators of health status (Gurunathan et al., 2022; Ortega et al., 2022).

A few studies have profiled differences between EV cargos from term and preterm pregnancies, suggesting their biomarkers and cargo can predict high risk pregnancy status. A recent illustration of this potential is a new multiple microarray analyzer for identifying surface markers on plasma EV that predict preterm delivery and preeclampsia compared with term delivery controls in humans (Jørgensen et al., 2025). The present study aimed to assess and characterize the protein profiles in EV at parturition from the maternal plasma of dairy cows exposed to late-gestation heat stress. We hypothesized that heat stress during late gestation would alter the protein profile of EV at parturition in dairy cows.

Experiments were conducted over the summer months (July to October) of 2022 and 2023 at the University of Florida Dairy Unit (Hague, FL). The University of Florida Institutional Animal Care and Use Committee approved all procedures. An extended description of the animal care and experimental design of this study is reported in Casarotto et al. (2025). Multiparous pregnant Holstein cows (parity 1.3 ± 0.8) had lactation terminated per normal management procedures (i.e., dried-off) at 232 ± 5 d of gestation and were randomly assigned to 1 of 2 treatments (cooling or heat stress), blocked by their expected calving date and mature-equivalent milk production. Before enrollment, dams were managed identically, with access to the shade of a freestall barn, fans, and soakers during their lactation. Upon enrollment, cows were housed in the same sand-bedded freestall barns in separate adjacent pens, either with an active cooling system (**CL**; access to the shade of a barn plus forced ventilation via fans and water soakers over the feed lane) or heat stress (**HT**; access to the shade of a barn and natural ventilation, no active cooling devices) for the entire duration of their dry period of 54 ± 5 d. Sample collection was initiated in cows delivering female calves in each treatment, and the study was powered to address productive responses rather than EV abundance.

A 7-mL blood sample was collected from the dams (n = 17/treatment) by coccygeal vessel puncture into EDTA Vacutainers (Becton Dickinson, Franklin Lakes, NJ) within 2 h after parturition. Upon collection, samples were immediately placed in ice. Blood was centrifuged at 2,500 × *g* at 4°C for 20 min within 1 h after collection for plasma separation. After centrifugation, plasma samples were frozen at −80°C until analysis. After thawing the plasma samples at 4°C, precipitates were removed with 2 successive centrifugations at 3,000 × *g* at 4°C for 15 min. The EV were isolated from plasma via single-step size exclusion chromatography, using an automatic fraction collector, through the Izon qEV isolation platform using the 70 nm column for isolation (Izon Science, Christchurch, New Zealand). Preparations were checked for quality by transmission electronic microscopy and then quantified by nanoparticle tracking analysis before proteomic analysis. Vesicles had an average size ± SD of 118.6 ± 11.5 nm, with a concentration of 5.94E+10 ± 4.36E+10 particles/mL in the HT samples and 122.7 ± 7.2 nm with concentration of 4.92E+10 ± 5.12E+10 particles/mL in the CL.

For protein extraction, vesicles were disrupted in Laemmli buffer overnight at room temperature. Proteins (5 µL) were loaded on SDS-PAGE (short migration). Bands of gel were cut, and proteins were reduced with dithiothreitol (10 m*M*) for 30 min at 56°C and alkylated with iodoacetamide (final concentration 55 m*M*) for 45 min at room temperature in the dark. In-gel digestion was conducted with 50 m*M* ammonium bicarbonate (pH 8.0) overnight at 37°C with 50 ng trypsin (Promega) per sample. Peptides were extracted by 5% formic acid in water/acetonitrile (vol/vol). The supernatant and extracted tryptic peptides were dried and resuspended in 40 µL of 0.1% (vol/vol) formic acid and 2% (vol/vol) acetonitrile.

For MS, the samples were analyzed on a NanoElute LC system (Bruker Daltonic GmbH) coupled to a timsTOF Pro (Bruker Daltonic GmbH) equipped with a CaptiveSpray source. Peptides were separated on a 25 cm × 75 μm analytical column (maintained at 50°C, with 1.6-μm C18 beads with a packed emitter tip (IonOpticks, Australia) using a constant flow rate of 250 nL min^−1^. The multigradient steps began at 1 min from 5% to 13% buffer B (0.1% formic acid and 100% acetonitrile) over 19 min, then 19% over 26 min, then 22% over 30 min before increasing to 95% buffer B and sustained for 7 min. The mobile phase was then increased back to 98% buffer A (0.1% formic acid and 98% water) and sustained for 2 min. The timsTOF Pro was operated using a data-independent acquisition method using parallel accumulation-serial fragmentation (**DIA-PASEF**) with the following settings: MS survey scan of 100 to 1,700 *m*/*z*, with ion mobility range (1/k0) of 0.7 to 1.2 V·s/cm^2^. The trapped ion mobility spectrometry (TIMS) analyzer was operated in a 100% duty cycle with equal accumulation and ramp times of 100 ms each and a total cycle time estimated at 1.6 s. During DIA-PASEF MS/MS scan, precursors with *m*/*z* between 247 and 1,047 were defined with 32 ion mobility steps with an isolation window of 26 Da in each step, with a 1-Da overlap with neighboring windows. The collision energy for the DIA-PASEF scan was increased linearly from 59 eV at 1/k0 = 1.6 V·s/cm^2^ to 20 eV at 1/k0 = 0.6 V·s/cm^2^.

Biostatistical analysis was performed to identify the proteins using DIANN v.1.8.1 (Demichev et al., 2020) by matching peptides against the Swiss-Prot *Bos taurus* database containing 48,128 entries (version 2023; https://www.uniprot.org/). Proteins were filtered to eliminate spectra due to contaminants. The data were also compared with a contaminant database. The proteome identification was analyzed with a precursor and fragment mass tolerance of 20 ppm. Enzymatic cleavage rules were set to trypsin digestion (“after Arg and Lys, unless Pro follows directly after”), and no semi-enzymatic cleavage rules were allowed. The fixed modification was set to cysteine carbamidomethylation, and methionine oxidation was considered a potential modification. The identified proteins were filtered with a q-value <0.01. Descriptive and statistical analysis using DIANN results were performed with Shiny proteom_IC (https://github.com/MarjorieLeduc/Shiny_PROTEOM_IC/tree/main). A 2-sided, unpaired Welch's *t*-test was done on proteins showing at least 3 valid values in one group and at least 70% of valid values in the other group using log2 (label-free quantification intensity). The significance threshold was *P* ≤ 0.059, and proteins with a log 2-fold-change (**log2FC**; CL/HT) above 1.2 or below 0.66 were considered significantly affected. Enrichment analysis with the overrepresentation of biological processes and molecular function (Gene Ontology [**GO**]) and protein interaction was created with the STRING Consortium 2025 database (https://string-db.org) using the protein ID and their corresponding log2FC. The raw MS data and corresponding protein tables and analysis are available from the corresponding author upon reasonable request.

Animal parameters and environmental conditions were previously detailed in Casarotto et al. (2025). Rectal temperatures and respiration rates averaged 39.1°C ± 0.2°C versus 38.5°C ± 0.3°C and 74.2 ± 6.9 versus 56.6 ± 8.7 breaths per minute for HT and CL cows, respectively, confirming a heat stress effect during the dry period. Extracellular vesicles from bovine plasma detected, including several proteins, were significantly affected when comparing CL and HT. A total of 684 proteins were identified by MS, with 20 proteins meeting the significance threshold of *P* ≤ 0.059 in CL compared with the HT treatment. Proteins increased in the CL group included laminin subunit gamma 1 (log2FC = 0.65; *P* = 0.001; Table 1), α 2 (log2FC = 0.67; *P* = 0.006) and β 1 (log2FC = 0.44; *P* = 0.056); transferrin receptor protein 1 (log2FC = 0.64; *P* = 0.028); collagen IV (log2FC = 0.48; *P* = 0.037); fibrinogen α chain (log2FC = 0.66; *P* = 0.042), gamma-B chain (log2FC = 0.63; *P* = 0.051), and β chain (log2FC = 0.66; *P* = 0.054); von Willebrand factor (log2FC = 1.08; *P* = 0.055); and SPN protein (log2FC = 0.43; *P* = 0.059). Furthermore, in the CL group, the proteins less abundant included immunoglobulin domain lambda (log2FC = −1.0; *P* = 0.014) and heavy chains (log2FC = −1.22; *P* = 0.023); Hepatocyte growth factor (HGF) activator (log2FC = −0.58; *P* = 0.016); protein HP-20 homolog (log2FC = −0.58; *P* = 0.028); trafficking from ER to Golgi regulator (TFG) protein (log2FC = −0.98; *P* = 0.041); amine oxidase 3 (log2FC = −0.65; *P* = 0.046); collectin member 10 (log2FC = −0.95; *P* = 0.049). Two significant proteins, A0A3Q1NKM0 (log2FC = 0.49; *P* = 0.007) and A0A3Q1LVJ5 (log2FC = −0.77, *P* = 0.02), could not be identified as specific proteins.

**Table 1:** Extracellular vesicle proteins detected on maternal plasma at parturition from cows that were exposed to late-gestation HT (n = 16) or CL (n = 17) with the corresponding gene, log2 fold change (log2FC) quantified in CL vs. HT, *P*-value, and function of the protein^1^

Protein	Gene	log2FC	*P*-value	Function
Basal membrane and extracellular matrix				
Laminin subunit gamma 1	*LAMC1*	0.65	0.001	Cell migration; EMX disassembly; tissue development
Laminin subunit α 2	*LAMA2*	0.67	0.006	Cell migration and adhesion
Protein HP-20 homolog	*—*	−0.58	0.028	COL trimer
Collagen α-1(IV) chain	*COL4A1*	0.48	0.037	EMX component and organization
Collectin subfamily member 10	*COLEC10*	−0.95	0.049	COL trimer
Laminin subunit β 1	*LAMB1*	0.44	0.056	Cell adhesion
A0A3Q1NKM0	*—*	0.49	0.007	—
Procoagulator factors				
HGF activator	*HGFAC*	−0.58	0.016	EMX space; serine protease; blood coagulation
Fibrinogen α chain	*FGA*	0.66	0.042	Fibrinogen complex; blood coagulation; plaques aggregation
Amine oxidase 3	*AOC3*	−0.65	0.046	Oxidoreductase; metal binding (Ca, Co)
Fibrinogen gamma chain	*FGG*	0.63	0.051	Fibrinogen complex; blood coagulation; plaques aggregation
Fibrinogen β chain	*FGB*	0.66	0.054	Fibrinogen complex; blood coagulation; plaques aggregation
von Willebrand factor	*VWF*	1.08	0.055	Blood coagulation; cell adhesion
Immunity and cell receptors				
Ig-like domain-containing protein	*—*	−1.00	0.014	EMX space; immune response
Ig lambda chain variable region	*—*	−1.22	0.023	*—*
Transferrin receptor protein 1	*TFRC*	0.64	0.028	Iron receptor
TFG protein	*TFG*	−0.98	0.041	Vesicle transport
Ig heavy chain variable region	*—*	−0.68	0.053	Immunoglobulin complex
SPN protein	*SPN*	0.43	0.059	Cell surface receptor
A0A3Q1LVJ5	*—*	−0.77	0.025	*—*

1 A negative logFC indicates less abundance and positive indicates more abundance of the protein.

A STRING network of protein interactions with both functional and physical protein association with their corresponding log2FC is shown in Figure 1. Enrichment analysis with protein interaction has shown the proteins being grouped by molecular function, such as extracellular matrix structural constituent (GO:0005201), cell adhesion molecular binding (GO:0050839), structural molecular activity (GO:0005198), and signaling receptor binding (GO:00055102; Figure 2A). Proteins were also grouped by biological processes such as platelet activation (GO:0030168), blood coagulation (GO:0007596), blood coagulation/fibrin clot formation (GO:0072378), fibrinolysis (GO:0042730), and positive regulation of cell-cell adhesion (GO:0034116; Figure 2B).

**Figure 1:**
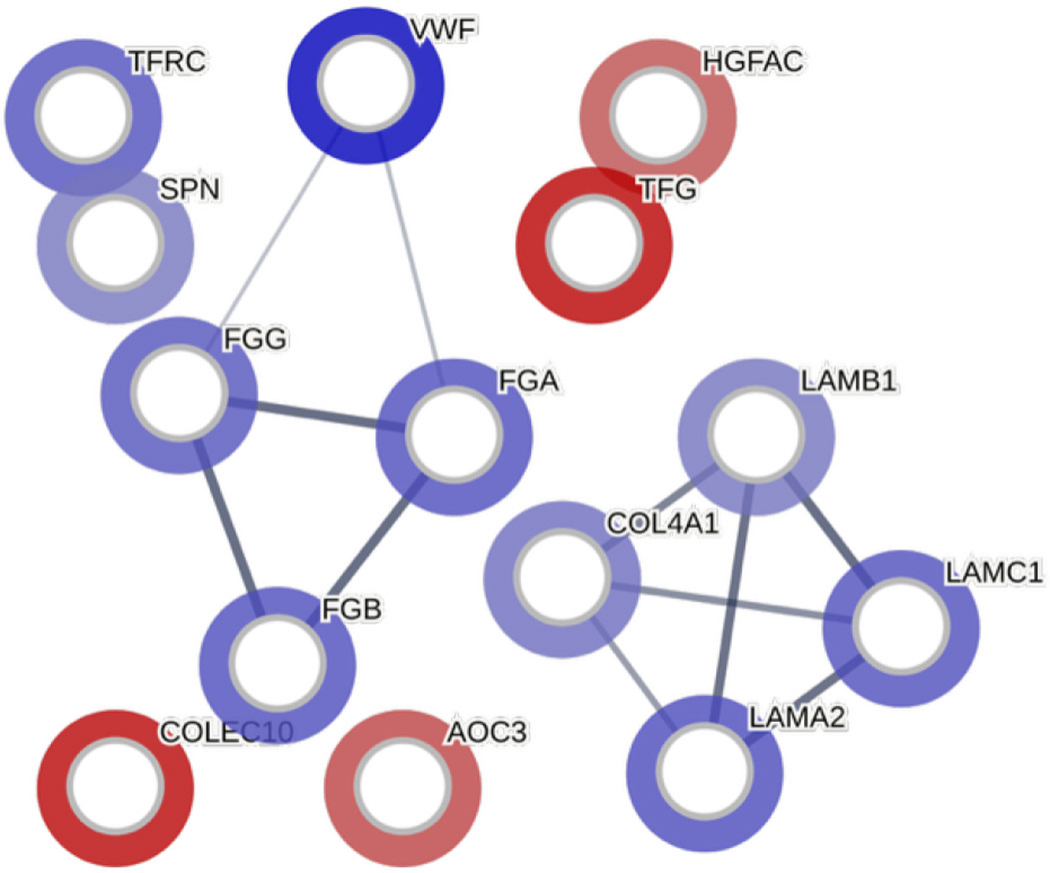
A STRING network of protein interaction with indication of both functional and physical protein association. Colors indicate the protein corresponding to log2FC, with blue indicating more abundant protein in CL compared with HT and red indicating less abundant proteins. Line thickness represents the strength of the data supporting the protein interaction. Figure created with the STRING Consortium 2025 database (https://string-db.org).

**Figure 2:**
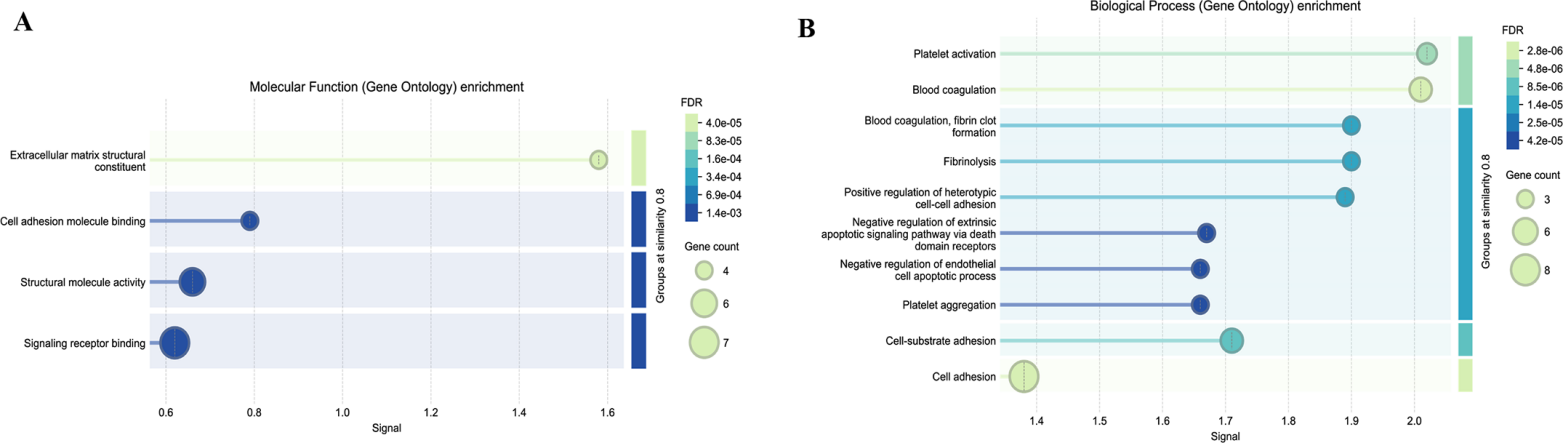
Functional enrichment analysis of EV proteins detected on maternal plasma at parturition from cows that were exposed to late-gestation heat stress (HT; n = 16) or cooling (CL; n = 17), with the corresponding gene count, false discovery rate (FDR), and the strength signal within the pathway of biological processes (A) and molecular function (B). Figure created with STRING Consortium 2025 database (https://string-db.org).

In this study, we identified 684 proteins in the EV circulating in the maternal plasma around parturition whose abundance was affected by the environmental temperature cows were exposed to during late gestation. Identifying and understanding the functions of those proteins carried by the EV are of relevance to assessing maternal health during pregnancy (Zhang et al., 2020). Most proteins identified as being affected by HT in late gestation could be categorized based on their functionality. These categories include constituents of the basement membrane and extracellular matrix (such as LAMC1, LAMA2, protein HP-20, COL4A1, COLEC10, and LAMB1), procoagulant factors (including FGA, HGFAC, AOC3, FGG, FGB, and VWF), immune-related factors (represented by immunoglobulin regions) and cell surface receptors (such as TFRC, TFG, and SPN). These proteins were present in the EV found in maternal blood circulation after parturition and may be involved in the transition from gestation to parturition and the altered physiological state of reproductive tissues such as the uterus and mammary gland.

Laminins are a family of glycoproteins that serve as key components of the basement membrane, playing a crucial role in cell differentiation, adhesion, and migration (Aumailley, 2013). After parturition, laminins are vital for uterine involution, tissue repair, and overall maternal recovery. These extracellular matrix (**EMX**) proteins influence cell adhesion, migration, and differentiation, which are essential for tissue remodeling and healing in the postpartum period (Rossi et al., 2025). Collagens are another important component of the EMX and are found in various tissues, including the placenta of dairy cows. A recent study has shown that exposure to heat stress during late gestation can significantly affect gene expression and methylation profiles regulating the structural integrity of the placenta, such as structural proteins (e.g., collagens and elastin), adhesion glycoproteins (e.g., fibronectin and laminin), and proteoglycans (e.g., syndecan), which are significantly downregulated compared with placentas from cows with access to active cooling in late gestation (Casarotto et al., 2025). We observed differences in the same proteins being carried by the maternal EV as the previous study reported in gene expression, with the COL4 protein in maternal circulation, as well as changes in proteins responsible for EMX degradation and proteases, such as HP20-homolog (Zhu et al., 2023) and COLEC10 (Wyatt and Crawford, 2021). The over-secretion of EMX proteins, coupled with a decrease in the secretion of proteins responsible for degrading the EMX in the CL cows, suggests that a more effective healing process occurred in the CL compared with the HT animals. This imbalance may favor tissue repair and regeneration, highlighting the potential differences in reproductive tissue response between these 2 groups during the transition period after partition. Therefore, these proteins may have the potential to be used as biomarkers of uterine involution and maternal recovery.

Extracellular vesicles can also carry procoagulants and support coagulation, transporting the protein complexes to the tissue for the coagulation cascade (Tripisciano et al., 2017). In humans, those procoagulant factors are crucial after parturition, regulating coagulation to reduce blood loss during and after delivery (Bardan et al., 2024). Although ruminants are less prone to bleeding at parturition due to the epitheliochorial placenta, the process of parturition is influenced by both extracellular EV and coagulation factors. Extracellular vesicles, particularly those derived from bovine placental tissue, play a crucial role in feto-maternal communication. Coagulation factors, including fibrinogen, prothrombin, and factor VII, exhibit changes in activity during pregnancy, whereas factors VIII and IX show a significant increase around the time of delivery (Kisker et al., 1981; Galli et al., 2024). In the current study, we identified proteins regulating coagulation as being affected by late-gestation heat stress. For example, fibrinogens and von Willebrand factor were more abundant in CL EV relative to HT. The CL treatment resulted in less abundance of HGF, and previous studies indicate that HGF increases in blood plasma and liver following hepatic injury, playing a crucial role in liver, kidney, lung, and stomach regeneration (Miyazawa, 2010). Skibiel (2024) reported that moderate HT before parturition time, appears to downregulate numerous hepatic genes and proteins involved in mitochondrial metabolism, which may contribute to oxidative stress, energy deficits, and impaired ability of heat-stressed cows to partition nutrients for milk synthesis in the subsequent lactation. The detection of dysregulated protein secretion in the circulation of cows, carried by EV at the time of parturition, may help us to understand how late-gestation heat stress affects the maternal response to parturition and subsequent recovery. Late-gestation heat stress may compromise maternal recovery and make cows more susceptible to postpartum health disorders (Molinari et al., 2022, 2023).

Last, a reduction in the immunoglobulin chains present in the EV cargo was observed in the HT cows compared with CL. Additionally, changes in cell surface receptors were noted. These findings are particularly intriguing because it would have been expected for the HT animals to exhibit a decrease in immunoglobulins, not the CL, which aligns with the known lower concentration of immunoglobulins in cow colostrum and being immunosuppressed at the time of parturition. However, the impact of heat stress on colostrum quality remains a topic of controversy. Some studies indicate that colostrum from cows exposed to HT contains lower concentrations of IgG and IgA (Nardone et al., 1997; Adin et al., 2009; Seyed Almoosavi et al., 2021), whereas others find no difference in IgG concentrations or even report an increased concentration of immunoglobulins (Monteiro et al., 2014; Skibiel et al., 2017). On the other hand, the current observation of reduced immunoglobulin load in CL dams might suggest a diminished demand for immune function in the CL cows, as studies have indicated that HT during late gestation is associated with a weakened the immune response. As a result, the ability of HT cows to recover effectively after giving birth is compromised, which can hinder the smooth transition into lactation and even subsequent reproductive competence. This weakened immune function could lead to increased susceptibility to infections and other health issues during a critical time for both the cow and her calf, which is consistent with previous reports (Dahl et al., 2016; Ouellet et al., 2020; Molinari et al., 2023).

In conclusion, this study demonstrates that late-gestation heat stress induces substantial changes in the protein cargo of maternal EV at parturition, particularly in proteins associated with extracellular matrix remodeling, coagulation, and immune responses. These alterations may compromise maternal recovery and immune function during the transition to lactation, potentially increasing susceptibility to postpartum health disorders. The identified EV proteins may serve as biomarkers for assessing maternal adaptation and recovery, emphasizing the importance of environmental management during the dry period to safeguard dairy cow health and productivity. We acknowledge several limitations in this study. The large number of proteins assessed and the exploratory nature of our analysis necessitated the use of a liberal significance threshold (*P* ≤ 0.059), increasing the risk of false positives due to multiple testing. Therefore, these findings should be interpreted as preliminary and hypothesis-generating, requiring confirmation in future independent validation studies.

## References

[bib1] Adin G., Gelman A., Solomon R., Flamenbaum I., Nikbachat M., Yosef E., Zenou A., Shamay A., Feuermann Y., Mabjeesh S.J., Miron J. (2009). Effects of cooling dry cows under heat load conditions on mammary gland enzymatic activity, intake of food and water, and performance during the dry period and after parturition. Livest. Sci..

[bib2] Al-Dossary A.A., Strehler E.E., Martin-DeLeon P.A. (2013). Expression and secretion of plasma membrane Ca^2+^-ATPase 4a (PMCA4a) during murine estrus: Association with oviductal exosomes and uptake in sperm. PLoS One.

[bib3] Almiñana C., Corbin E., Tsikis G., Alcântara-Neto A.S., Labas V., Reynaud K., Galio L., Uzbekov R., Garanina A.S., Druart X., Mermillod P. (2017). Oviduct extracellular vesicles protein content and their role during oviduct–embryo cross-talk. Reproduction.

[bib4] Aumailley M. (2013). The laminin family. Cell Adh. Migr..

[bib5] Bardan C.R., Ioniță I., Iordache M., Călămar-Popovici D., Todorescu V., Popescu R., Bernad B.C., Bardan R., Bernad E.S. (2024). Epigenetic biomarkers in thrombophilia-related pregnancy complications: mechanisms, diagnostic potential, and therapeutic implications: A narrative review. Int. J. Mol. Sci..

[bib6] Bernabucci U., Lacetera N., Baumgard L.H., Rhoads R.P., Ronchi B., Nardone A. (2010). Metabolic and hormonal acclimation to heat stress in domesticated ruminants. Animal.

[bib7] Burns G., Brooks K., Wildung M., Navakanitworakul R., Christenson L.K., Spencer T.E. (2014). Extracellular vesicles in luminal fluid of the ovine uterus. PLoS One.

[bib8] Casarotto L.T., Jones H.N., Chavatte-Palmer P., Laporta J., Peñagaricano F., Ouellet V., Bromfield J., Dahl G.E. (2025). Late-gestation heat stress alters placental structure and function in multiparous dairy cows. J. Dairy Sci..

[bib9] Collier R.J., Baumgard L.H., Zimbelman R.B., Xiao Y. (2019). Heat stress: Physiology of acclimation and adaptation. Anim. Front..

[bib10] Collier R.J., Renquist B.J., Xiao Y. (2017). A 100-Year Review: Stress physiology including heat stress. J. Dairy Sci..

[bib11] da Silveira J.C., Veeramachaneni D.N.R., Winger Q.A., Carnevale E.M., Bouma G.J. (2012). Cell-secreted vesicles in equine ovarian follicular fluid contain miRNAs and proteins: A possible new form of cell communication within the ovarian follicle. Biol. Reprod..

[bib12] Dahl G.E., Tao S., Monteiro A.P.A. (2016). Effects of late-gestation heat stress on immunity and performance of calves. J. Dairy Sci..

[bib13] Das M., Kale V. (2020). Extracellular vesicles: Mediators of embryo-maternal crosstalk during pregnancy and a new weapon to fight against infertility. Eur. J. Cell Biol..

[bib14] Das R., Sailo L., Verma N., Bharti P., Saikia J., Imtiwati, Kumar R. (2016). Impact of heat stress on health and performance of dairy animals: A review. Vet. World.

[bib15] Demichev V., Messner C.B., Vernardis S.I., Lilley K.S., Ralser M. (2020). DIA-NN: Neural networks and interference correction enable deep proteome coverage in high throughput. Nat. Methods.

[bib16] De Rensis F., Scaramuzzi R.J. (2003). Heat stress and seasonal effects on reproduction in the dairy cow—A review. Theriogenology.

[bib17] Galli J., Almiñana C., Wiesendanger M., Schuler G., Kowalewski M.P., Klisch K. (2024). Bovine placental extracellular vesicles carry the fusogenic syncytin BERV-K1. Theriogenology.

[bib18] Gurunathan S., Kang M.-H., Song H., Kim N.H., Kim J.-H. (2022). The role of extracellular vesicles in animal reproduction and diseases. J. Anim. Sci. Biotechnol..

[bib19] Jørgensen M.M., Bæk R., Sloth J.K., Sammour R., Sharabi-Nov A., Vatish M., Meiri H., Sammar M. (2025). A novel multiple marker microarray analyzer and methodology to predict major obstetric syndromes using surface markers of circulating extracellular vesicles from maternal plasma. Acta Obstet. Gynecol. Scand..

[bib20] Kalluri R., LeBleu V.S. (2020). The biology, function, and biomedical applications of exosomes. Science.

[bib21] Kisker C.T., Robillard J.E., Clarke W.R. (1981). Development of blood coagulation—A fetal lamb model. Pediatr. Res..

[bib22] Koh Y.Q., Peiris H.N., Vaswani K., Meier S., Burke C.R., Macdonald K.A., Roche J.R., Almughlliq F., Arachchige B.J., Reed S., Mitchell M.D. (2017). Characterization of exosomes from body fluids of dairy cows. J. Anim. Sci..

[bib23] Mellisho E.A., Velásquez A.E., Nuñez M.J., Cabezas J.G., Cueto J.A., Fader C., Castro F.O., Rodríguez-Álvarez L. (2017). Identification and characteristics of extracellular vesicles from bovine blastocysts produced in vitro. PLoS One.

[bib24] Miyazawa K. (2010). Hepatocyte growth factor activator (HGFA): A serine protease that links tissue injury to activation of hepatocyte growth factor. FEBS J..

[bib25] Molinari P.C.C., Dahl G.E., Sheldon I.M., Bromfield J.J. (2022). Effect of calving season on metritis incidence and bacterial content of the vagina in dairy cows. Theriogenology.

[bib26] Molinari P.C.C., Davidson B.D., Laporta J., Dahl G.E., Sheldon I.M., Bromfield J.J. (2023). Prepartum heat stress in dairy cows increases postpartum inflammatory responses in blood of lactating dairy cows. J. Dairy Sci..

[bib27] Monteiro A.P.A., Tao S., Thompson I.M., Dahl G.E. (2014). Effect of heat stress during late gestation on immune function and growth performance of calves: Isolation of altered colostral and calf factors. J. Dairy Sci..

[bib28] Nakahara A., Nair S., Ormazabal V., Elfeky O., Garvey C.E., Longo S., Salomon C. (2020). Circulating placental extracellular vesicles and their potential roles during pregnancy. Ochsner J..

[bib29] Nardone A., Lacetera N., Bernabucci U., Ronchi B. (1997). Composition of colostrum from dairy heifers exposed to high air temperatures during late pregnancy and the early postpartum period. J. Dairy Sci..

[bib30] Ng Y.H., Rome S., Jalabert A., Forterre A., Singh H., Hincks C.L., Salamonsen L.A. (2013). Endometrial exosomes/microvesicles in the uterine microenvironment: A new paradigm for embryo-endometrial cross talk at implantation. PLoS One.

[bib31] Ortega M.A., Fraile-Martínez O., García-Montero C., Sáez M.A., Álvarez-Mon M.A., Torres-Carranza D., Álvarez-Mon M., Bujan J., García-Honduvilla N., Bravo C., Guijarro L.G., De León-Luis J.A. (2022). The pivotal role of the placenta in normal and pathological pregnancies: A focus on preeclampsia, fetal growth restriction, and maternal chronic venous disease. Cells.

[bib32] Ouellet V., Laporta J., Dahl G.E. (2020). Late gestation heat stress in dairy cows: Effects on dam and daughter. Theriogenology.

[bib33] Rossi F., Luppi S., Fejza A., Giolo E., Ricci G., Andreuzzi E. (2025). Extracellular matrix and pregnancy: functions and opportunities caught in the net. Reprod. Biol. Endocrinol..

[bib34] Roth Z. (2020). Influence of heat stress on reproduction in dairy cows-physiological and practical aspects. J. Anim. Sci..

[bib35] Seyed Almoosavi S.M.M., Ghoorchi T., Naserian A.A., Khanaki H., Drackley J.K., Ghaffari M.H. (2021). Effects of late-gestation heat stress independent of reduced feed intake on colostrum, metabolism at calving, and milk yield in early lactation of dairy cows. J. Dairy Sci..

[bib36] Skibiel A.L. (2024). Hepatic mitochondrial bioenergetics and metabolism across lactation and in response to heat stress in dairy cows. JDS Commun..

[bib37] Skibiel A.L., Fabris T.F., Corrá F.N., Torres Y.M., McLean D.J., Chapman J.D., Kirk D.J., Dahl G.E., Laporta J. (2017). Effects of feeding an immunomodulatory supplement to heat-stressed or actively cooled cows during late gestation on postnatal immunity, health, and growth of calves. J. Dairy Sci..

[bib38] Soliman S.M., El-Saadony M.T., Saad A., Mosa W.F.A., Khalil F.M.A., Ahmed A.E., Mohammed D.M., Manasar M.M., Farag M.R., Alagawany M., Salem H.M. (2025). The impacts of thermal stress on dairy cattle physiology, metabolism, health, and performance: a comprehensive review. Ann. Anim. Sci..

[bib39] Tao S., Dahl G.E. (2013). Invited review: Heat stress effects during late gestation on dry cows and their calves. J. Dairy Sci..

[bib40] Tao S., Orellana Rivas R.M., Marins T.N., Chen Y.-C., Gao J., Bernard J.K. (2020). Impact of heat stress on lactational performance of dairy cows. Theriogenology.

[bib41] Tersigni C., Furqan Bari M., Cai S., Zhang W., Kandzija N., Buchan A., Miranda F., Di Simone N., Redman C.W., Bastie C., Vatish M. (2022). Syncytiotrophoblast-derived extracellular vesicles carry apolipoprotein-E and affect lipid synthesis of liver cells in vitro. J. Cell. Mol. Med..

[bib42] Tripisciano C., Weiss R., Eichhorn T., Spittler A., Heuser T., Fischer M.B., Weber V. (2017). Different potential of extracellular vesicles to support thrombin generation: Contributions of phosphatidylserine, tissue factor, and cellular origin. Sci. Rep..

[bib43] West J.W. (2003). Effects of heat-stress on production in dairy cattle. J. Dairy Sci..

[bib44] Wyatt R.A., Crawford B.D. (2021). Post-translational activation of Mmp2 correlates with patterns of active collagen degradation during the development of the zebrafish tail. Dev. Biol..

[bib45] Yang C., Song G., Lim W. (2019). Effects of extracellular vesicles on placentation and pregnancy disorders. Reproduction.

[bib46] Zhang J., Li H., Fan B., Xu W., Zhang X. (2020). Extracellular vesicles in normal pregnancy and pregnancy-related diseases. J. Cell. Mol. Med..

[bib47] Zhu Y., Shigeyoshi K., Hayakawa Y., Fujiwara S., Kishida M., Ohki H., Horibe T., Shionyu M., Mizukami T., Hasegawa M. (2023). Acceleration of protein degradation by 20s proteasome-binding peptides generated by in vitro artificial evolution. Int. J. Mol. Sci..

